# Supplementary figures and images for: Early centralized isolation strategy for all confirmed cases of COVID-19 remains a core intervention to disrupt the pandemic spreading significantly
Source: PLoS One. 2021 Jul 15;16(7):e0254012. doi: 10.1371/journal.pone.0254012 (PMC8282022; doi:10.1371/journal.pone.0254012)

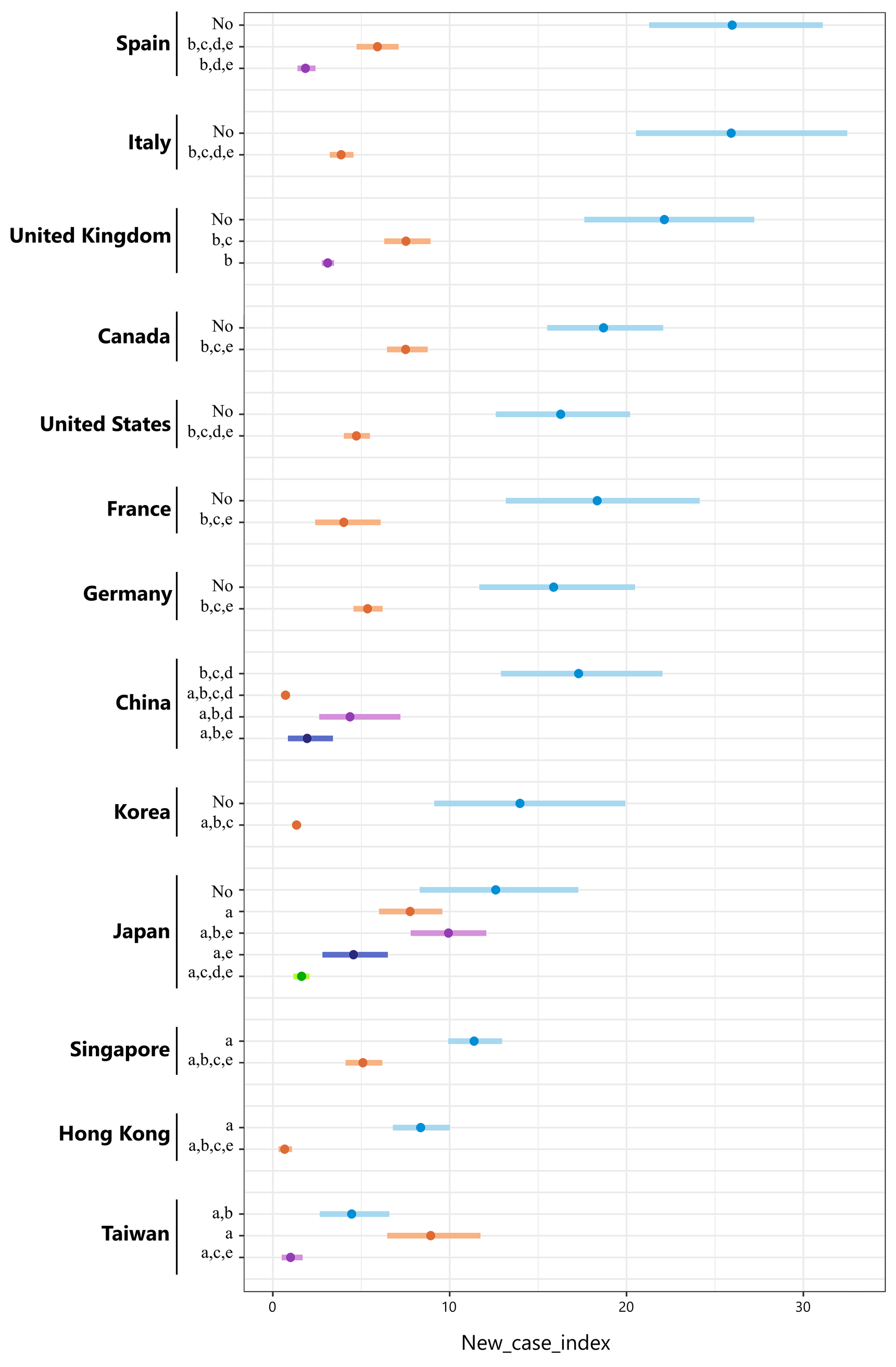

Supplement: S1 Fig — (TIF) [file pone.0254012.s001.tif]

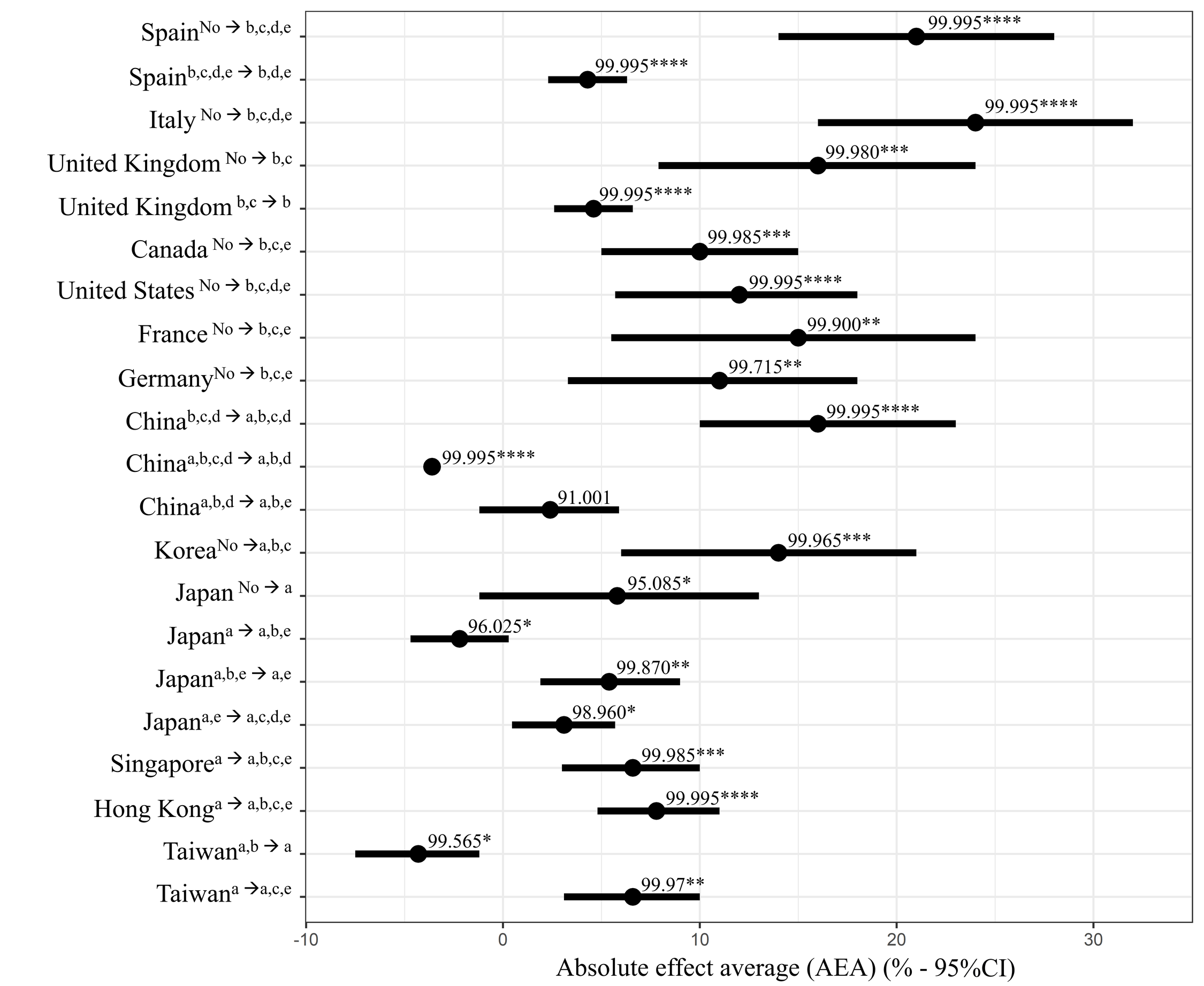

Supplement: S2 Fig — (Posterior probability of a causal effect; * < 0.05, ** < 0.01, *** < 0.001, **** < 0.0001). (TIF) [file pone.0254012.s002.tif]
