# Supplementary material for: Early centralized isolation strategy for all confirmed cases of COVID-19 remains a core intervention to disrupt the pandemic spreading significantly
Source: PLoS One. 2021 Jul 15;16(7):e0254012. doi: 10.1371/journal.pone.0254012 (PMC8282022; doi:10.1371/journal.pone.0254012)
Supplement: S1 Table — (DOCX) [file pone.0254012.s003.docx]

S1 Table: 13 countries with the highest percentage of days having the highest number of daily confirmed cases

| **ID** | **Country** | **N (days)** | **Percentage of days having the highest number of daily confirmed cases** |
| --- | --- | --- | --- |
| **1** | China | 70 | 100.00% |
| **2** | South Korea | 70 | 100.00% |
| **3** | United State | 70 | 100.00% |
| **4** | France | 68 | 97.14% |
| **5** | Germany | 65 | 92.86% |
| **6** | Italy | 61 | 87.14% |
| **7** | United Kingdom | 61 | 87.14% |
| **8** | Japan | 60 | 85.71% |
| **9** | Canada | 53 | 75.71% |
| **10** | Singapore | 51 | 72.86% |
| **11** | Hong Kong | 48 | 68.57% |
| **12** | Spain | 47 | 67.14% |
| **13** | Taiwan | 43 | 61.43% |
