# Supplementary material for: Early centralized isolation strategy for all confirmed cases of COVID-19 remains a core intervention to disrupt the pandemic spreading significantly
Source: PLoS One. 2021 Jul 15;16(7):e0254012. doi: 10.1371/journal.pone.0254012 (PMC8282022; doi:10.1371/journal.pone.0254012)
Supplement: S2 Table — (DOCX) [file pone.0254012.s004.docx]

S2 Table: The average value of the ratio of daily new case between the 5 countries and Sweden

| Day | Spain | Italy | United Kingdom | France | Germany | Mean of daily new cases of 5 countries | New cases of Sweden | The ratio of daily new cases between 5 countries and Sweden |
| --- | --- | --- | --- | --- | --- | --- | --- | --- |
| 1* | 400 | 240 | 342 | 296 | 241 | 303.8 | 71 | 4.28 |
| 2 | 622 | 566 | 0 | 177 | 136 | 300.2 | 69 | 4.35 |
| 3 | 582 | 342 | 403 | 83 | 281 | 338.2 | 83 | 4.07 |
| 4 | 0 | 466 | 407 | 575 | 451 | 379.8 | 119 | 3.19 |
| 5 | 2955 | 587 | 676 | 497 | 170 | 977 | 145 | 6.74 |
| 6 | 1159 | 769 | 63 | 0 | 1597 | 717.6 | 143 | 5.02 |
| 7 | 1407 | 778 | 1294 | 1380 | 910 | 1153.8 | 180 | 6.41 |
| 8 | 2144 | 1247 | 1035 | 808 | 1210 | 1288.8 | 135 | 9.55 |
| 9 | 1806 | 1492 | 665 | 30 | 1477 | 1094 | 118 | 9.27 |
| 10 | 2162 | 1797 | 967 | 2134 | 1985 | 1809 | 182 | 9.94 |
| 11 | 4053 | 977 | 1427 | 1019 | 3070 | 2109.2 | 230 | 9.17 |
| 12 | 2447 | 2313 | 1452 | 1391 | 2993 | 2119.2 | 314 | 6.75 |
| 13 | 4964 | 0 | 2129 | 1828 | 4528 | 2689.8 | 286 | 9.40 |
| 14 | 3394 | 5198 | 2885 | 1741 | 2365 | 3116.6 | 365 | 8.54 |
| 15 | 6368 | 3497 | 2546 | 1670 | 2660 | 3348.2 | 300 | 11.16 |
| 16 | 4749 | 3590 | 2433 | 1736 | 4183 | 3338.2 | 280 | 11.92 |
| 17 | 9630 | 3233 | 2619 | 3838 | 3930 | 4650 | 416 | 11.18 |
| 18 | 8271 | 3526 | 3009 | 2448 | 4337 | 4318.2 | 475 | 9.09 |
| 19 | 7933 | 4207 | 4324 | 2929 | 6615 | 5201.6 | 486 | 10.70 |
| 20 | 7516 | 5322 | 4244 | 3922 | 6933 | 5587.4 | 554 | 10.09 |
| 21 | 6875 | 5986 |  | 3809 | 6824 | 5873.5 | 601 | 9.77 |
| 22 |  | 6557 |  | 4611 |  | 5584 | 357 | 15.64 |
| 23 |  | 5560 |  | 2599 |  | 4079.5 | 340 | 12.00 |
| 24 |  | 4789 |  | 4376 |  | 4582.5 | 389 | 11.78 |
| 25 |  |  |  | 7578 |  | 7578 | 738 | 10.27 |
|  | | | | | | | |  |
| The average value of the ratio of daily new case between the 5 countries and Sweden | | | | | | | | **8.47** |

* “Day 1” was on March, 14^th^ 2020 when Sweden passed 900 confirmed cases. This was also when Sweden's data started to change abnormally compared to the 5 European countries mentioned above. To estimate the ratio between the 5 countries and Sweden, we also took “Day 1” of the 5 countries mentioned above, which has passed 900 confirmed cases.
