# Supplementary material for: Early centralized isolation strategy for all confirmed cases of COVID-19 remains a core intervention to disrupt the pandemic spreading significantly
Source: PLoS One. 2021 Jul 15;16(7):e0254012. doi: 10.1371/journal.pone.0254012 (PMC8282022; doi:10.1371/journal.pone.0254012)
Supplement: S3 Table — (DOCX) [file pone.0254012.s005.docx]

**S3 Table: Level of lock-down strategy and its definition**

| Lock-down strategy | Definition |
| --- | --- |
| Centralized isolation of all confirmed cases | Centralized isolation of all patients with positive COVID-19 |
| Closure of schools | Closure of schools and universities |
| Closure of public areas | Closure of public areas, such as entertainment facilities, bars, karaoke or massage parlors, fitness centers, spas, and beauty clinics |
| Closure of cities | - Closure of non-essential business activities; except for food grocers, pharmacies, urgent medical circumstances, and essential places of employment  - Suspension of entry of all foreigners  - Suspension of public modes of transportation  - Request for citizens to stay at home |
| Closure of borders | Closure of national borders |
