# Supplementary material for: Early centralized isolation strategy for all confirmed cases of COVID-19 remains a core intervention to disrupt the pandemic spreading significantly
Source: PLoS One. 2021 Jul 15;16(7):e0254012. doi: 10.1371/journal.pone.0254012 (PMC8282022; doi:10.1371/journal.pone.0254012)
Supplement: S4 Table — (DOCX) [file pone.0254012.s006.docx]

**S4 Table: Downtime of the lock-down strategy as of May 11**

| **Country** | **Reopened** | **Date** |
| --- | --- | --- |
| Spain | Public areas | 13 April |
| Italy | Open borders | 4 May |
| UK | Public areas | 11 April |
| Germany | Public areas | 20 April |
| China | City | 8 April |
| South Korea | Public areas | 6 May |
| Japan | School | 6 April |
| Taiwan | School | 25 February |
