# Supplementary material for: Early centralized isolation strategy for all confirmed cases of COVID-19 remains a core intervention to disrupt the pandemic spreading significantly
Source: PLoS One. 2021 Jul 15;16(7):e0254012. doi: 10.1371/journal.pone.0254012 (PMC8282022; doi:10.1371/journal.pone.0254012)
Supplement: S6 Table — (DOCX) [file pone.0254012.s008.docx]

S6 Table: Insignificant data changes due to the fluctuation of time between testing and reporting in official statistics

| **Country** | **Absolute effect OR (95% CI)** | | | | |
| --- | --- | --- | --- | --- | --- |
|  | **Day 13th** | **Day 12th (-1)** | **Day 11th (–2)** | **Day 10th (-3)** | **Day 9th (-4)** |
| Spain^No 🡪 b,c,d,e^ | 21 (14, 28)**** | 21 (14, 29)**** | 21 (14, 29)**** | 21 (13, 29)**** | 21 (13, 29)**** |
| Spain^b,c,d,e 🡪 b,d,e^ | 4.3 (2.3, 6.3)**** | 4.6 (2.5, 6.6)**** | 4.9 (2.8, 7.1)*** | 5.6 (3.5, 7.8)**** | 6 (3.6, 8.4)**** |
| Italy ^No 🡪 b,c,d,e^ | 24 (16, 32)**** | 24 (16, 33)**** | 25 (17, 33)**** | 25 (17, 34)**** | 25 (17, 34)**** |
| United Kingdom ^No 🡪 b,c^ | 16 (7.9, 24)*** | 16 (7.5, 24)*** | 16 (7.3, 24)*** | 15 (7, 24)*** | 15 (6.8, 24)*** |
| United Kingdom ^b,c 🡪 b^ | 4.6 (2.6, 6.6)**** | 5.0 (2.9, 7.1)**** | 5.6 (3.3, 8)**** | 5.8 (3.6, 8.1)**** | 6.2 (4, 8.4)**** |
| Canada ^No 🡪 b,c,e^ | 10 (5, 15)*** | 9.9 (4.9, 15)*** | 9.7 (4.6, 15)*** | 9.8 (4.7, 15)*** | 9.6 (4.4, 15)*** |
| United States ^No 🡪 b,c,d,e^ | 12 (5.7, 18)**** | 12 (5.5, 18)*** | 12 (5.4, 18)*** | 12 (5.2, 18)*** | 12 (5.1, 18)*** |
| France ^No 🡪 b,c,e^ | 15 (5.5, 24)** | 15 (5.5, 24)** | 15 (5.5, 24)*** | 15 (5.7, 24)*** | 15 (5.6, 24)** |
| Germany^No 🡪 b,c,e^ | 11 (3.3, 18)** | 11 (3.3, 18)** | 11 (3.1, 18)** | 10 (2.7, 18)** | 10 (2.5, 18)** |
| China^b,c,d 🡪 a,b,c,d^ | 16 (10, 23)**** | 17 (11, 23)**** | 17 (11, 24)**** | 18 (12, 24)**** | 18 (12, 24)**** |
| China^a,b,c,d 🡪 a,b,d^ | -3.6 (-3.2, -3.9)**** | -3.5 (-3.1, 3.9)**** | -3.4 (-3.9, 3)**** | -3.3 (-3.8, -2.8)**** | -1.7 (-2.7, -0.63)*** |
| China^a,b,d 🡪 a,b,e^ | 2.4 (-1.2, 5.9) | 2.4 (-1.1, 5.9) | 2.4 (-1.1, 5.8) | 2.3 (-1.2, 5.7) | -0.071 (-1.3, 1.2) |
| Korea^No 🡪a,b,c^ | 14 (6, 21)*** | 14 (6.3, 22)** | 14 (6.5, 22)*** | 15 (7, 22)*** | 15 (7.1, 23)*** |
| Japan ^No 🡪 a^ | 5.8 (-1.2, 13)* | 5.4 (-1.6, 13) | 5.2 (-2, 13) | 5.4 (-2.1, 13) | 5.2 (-2.4, 13) |
| Japan^a 🡪 a,b,e^ | -2.2 (-4.7, 0.29)* | -1.6 (-4.2, 1.6) | -1.5 (-4, 1.1) | -1.3 (-3.8, 1.2) | -0.53 (-2.9, 1.9) |
| Japan^a,b,e 🡪 a,e^ | 5.4 (1.9, 9)** | 4.9 (1.3, 8.4)** | 4.6 (1, 8.1)** | 4.1 (0.53, 7.7)* | 2.8 (-0.98, 6.5) |
| Japan^a,e 🡪 a,c,d,e^ | 3.1 (0.46, 5.7)* | 3.4 (0.88, 5.9)** | 3.6 (1.4, 5.9)** | 3.9 (1.6, 6.3)** | 4.8 (2.3, 7.5)*** |
| Singapore^a 🡪 a,b,c,e^ | 6.6 (3, 10)*** | 6.2 (2.6, 9.7)*** | 5.4 (1.9, 8.9)** | 5.3 (1.8, 8.7)** | 4.7 (1.3, 8.1)** |
| Hong Kong^a 🡪 a,b,c,e^ | 7.8 (4.8, 11)**** | 7.9 (4.9, 11)**** | 8 (5, 11)**** | 8 (5, 11)**** | 8 (5, 11)**** |
| Taiwan^a,b 🡪 a^ | -4.3 (-7.5, -1.2)* | -4.2 (-7.4, -1)** | -3.9 (-7.2, -0.71)** | -3.7 (-7, -0.39)* | -3.7 (-7, -0.35)* |
| Taiwan^a 🡪a,c,e^ | 6.6 (3.1, 10)** | 8.9 (4.5, 13)*** | 7.8 (4.2, 12)*** | 6.4 (3.8, 11)**** | 9.9 (5.4, 15)*** |
| * < 0.05 , ** < 0.01, *** < 0.001, **** < 0.0001 | | | | | |
