# Supplementary material for: Early centralized isolation strategy for all confirmed cases of COVID-19 remains a core intervention to disrupt the pandemic spreading significantly
Source: PLoS One. 2021 Jul 15;16(7):e0254012. doi: 10.1371/journal.pone.0254012 (PMC8282022; doi:10.1371/journal.pone.0254012)
Supplement: S8 Table — (DOCX) [file pone.0254012.s010.docx]

S8 Table: Absolute effect of government policies to reproduction rate with different timelines

| **Country** | **Absolute effect of the reproduction rate (95% CI)^p^** | | | | |
| --- | --- | --- | --- | --- | --- |
|  | **13 days** | **12 days (– 1)** | **11 days (– 2)** | **10 days (– 3)** | **9 days (– 4)** |
| Spain^No 🡪 bcde^ | -1.9 (-2.3, -1.5)**** | -1.9 (-2.3, -1.5)**** | -1.9 (-2.3, -1.5)**** | -1.9 (-2.3, -1.5)**** | -1.9 (-2.3, -1.5)**** |
| Spain^bcde 🡪 bde^ | -0.31 (-0.44, -0.19)**** | -0.35 (-0.49, -0.21)**** | -0.38 (-0.53, -0.23)**** | -0.42 (-0.59, -0.25)**** | -0.45 (-0.64, -0.27)**** |
| Italy ^No 🡪 bcde^ | -2.5 (-3.7, -1.4)**** | -2.6 (-3.8, -1.5)**** | -2.6 (-3.8, -1.5)**** | -2.7 (-3.9, -1.5)**** | -2.7 (-3.9, -1.6)**** |
| United Kingdom ^No 🡪 bc^ | -1.2 (-1.5, -0.99)**** | -1.2 (-1.5, -1)**** | -1.2 (-1.5, -1)**** | -1.2 (-1.5, -1)**** | -1.2 (-1.5, -0.99)**** |
| United Kingdom ^bc 🡪 b^ | -0.25 (-0.34, -0.16)**** | -0.27 (-0.37, -0.17)**** | -0.29 (-0.4, -0.18)**** | -0.32 (-0.44, -0.2)**** | -0.35 (-0.48, -0.21)**** |
| Canada ^No 🡪 bce^ | -1.3 (-1.4, -1.1)**** | -1.3 (-1.4, -1.2)**** | -1.3 (-1.4, -1.2)**** | -1.3 (-1.4, -1.2)**** | -1.3 (-1.4, -1.2)**** |
| United States ^No 🡪 bcde^ | -1.7 (-1.9, -1.4)**** | -1.7 (-1.9, -1.4)**** | -1.7 (-1.9, -1.4)**** | -1.7 (-1.9, -1.4)**** | -1.7 (-1.9, -1.4)**** |
| France ^No 🡪 bce^ | -1.7 (-2, -1.4)**** | -1.7 (-2, -1.4)**** | -1.7 (-2, -1.4)**** | -1.7 (-2, -1.4)**** | -1.7 (-2, -1.4)**** |
| Germany^No 🡪 bce^ | -1.6 (-1.9, -1.4)**** | -1.6 (-1.8, -1.4)**** | -1.6 (-1.8, -1.4)**** | -1.6-1.3 (-1.8, -1.4)**** | -1.6 (-1.8, -1.4)**** |
| China^bcd 🡪 abcd^ | -2 (-2.6, -1.5)**** | -2 (-2.5, -1.6)**** | -2.1 (-2.5, -1.6)**** | -2.1 (-2.6, -1.7)**** | -2.1 (-2.6, -1.7)**** |
| China^abcd 🡪 abd^ | 0.5 (0.43, 0.56)**** | 0.52 (0.41, 0.63)**** | 0.52 (0.41, 0.6)**** | 0.5 (0.38, 0.62)**** | 0.47 (0.32, 0.61)**** |
| China^abd 🡪 abe^ | -0.4 (-0.52, -0.29)**** | -0.5 (-0.55, -0.45)**** | -0.49 (-0.54, -0.44)**** | -0.47 (-0.52, -0.42)**** | -0.43 (-0.48, -0.38)**** |
| Korea^No 🡪abc^ | -1.1 (-1.7, -0.6)**** | -1.2 (-1.7, -0.64)**** | -1.2 (-1.8, -0.68)**** | -1.3 (-1.8, -0.74)**** | -1.3 (-1.8, -0.79)**** |
| Japan ^No 🡪 a^ | -0.093 (-0.23, 0.036)^0.082^ | -0.11 (-0.24, 0.02)* | -0.13 (-0.26, 0.004)* | -0.14 (-0.27, -0.013)* | -0.15 (-0.28, -0.025)** |
| Japan^a 🡪 abe^ | 0.52 (0.4, 0.64)**** | 0.41 (0.3, 0.52)**** | 0.41 (0.29, 0.52)**** | 0.4 (0.29, 0.51)**** | 0.38 (0.27, 0.5)**** |
| Japan^abe 🡪 ae^ | -0.68 (-0.8, -0.56)**** | -0.78 (-0.94, -0.61)**** | -0.73 (-0.9, -0.57)**** | -0.68 (-0.86, -0.5)**** | -0.62 (-0.81, -0.42)**** |
| Japan^ae 🡪 acde^ | -0.57 (-0.82, -0.33)**** | -0.35 (-0.48, -0.24)**** | -0.38 (-0.51, -0.26)**** | -0.42 (-0.56, -0.28)**** | -0.46 (-0.62, -0.32)**** |
| Singapore^a 🡪 abce^ | -0.43 (-0.61, -0.24)**** | -0.39 (-0.57, -0.21)*** | -0.35 (-0.52, -0.17)**** | -0.3 (-0.48, -0.13)**** | -0.26 (-0.42, -0.086)** |
| Hong Kong^a 🡪 abce^ | -0.49 (-0.67, -0.32)**** | -0.51 (-0.68, -0.35)**** | -0.53 (-0.69, -0.36)**** | -0.54 (-0.7, -0.39)**** | -0.56 (-0.71, -0.4)**** |
| Taiwan^ab 🡪 a^ | -0.38 (-0.43, -0.33)**** | -0.36 (-0.41, -0.31)**** | -0.33 (-0.39, -0.28)**** | -0.31 (-0.37, -0.26)**** | -0.29 (-0.34, -0.24)**** |
| Taiwan^a 🡪ace^ | -0.31 (-0.38, -0.25)**** | -0.39 (-0.53, -0.26)**** | -0.31 (-0.37, -0.26)**** | -0.32 (-0.38, -0.27)**** | -0.41 (-0.55, -0.28)**** |
| * < 0.05 ** < 0.01 *** < 0.001 **** < 0.0001 | | | | | |
