# Supplementary material for: Early centralized isolation strategy for all confirmed cases of COVID-19 remains a core intervention to disrupt the pandemic spreading significantly
Source: PLoS One. 2021 Jul 15;16(7):e0254012. doi: 10.1371/journal.pone.0254012 (PMC8282022; doi:10.1371/journal.pone.0254012)
Supplement: Attachment — Submitted filename: Review_report.pdf [file pone.0254012.s012.pdf]

# Review report

Manuscript title:

**Early centralized isolation strategy for all confirmed cases of COVID-19 remains a core intervention to disrupt the pandemic spreading significantly**

*Manuscript Number:* **PONE-D-20-33060R1**

**Summary**

This paper aims to show that the early centralized isolation is the major factor out of five control measures in disrupting the pandemic's spread. In order to assess the impact, a number of metrics has been used: new case indices, absolute effect average, absolute effect cumulative and relative effect average, posterior probability of a casual effect.

**Overall remarks**

The paper is well-written and well-organized. The main objective is clearly defined. However, the way of achieving the objective is not sufficient. In particular, the empirical analysis is not enough to reach the conclusion. Authors should incorporate some important metrics which are widely used to measure the spread of infectious disease. I believe that the authors can improve the paper by taking into account the suggestions provided below.

**Suggestions for improvement:**

1. We know that *reproduction rate* is statistically very important metric that measures the ability of any disease to spread. It would be great if authors can analyze *time-varying reproduction number* (R(t)) in pre-intervention and post-intervention periods.

2. One of the key indicators of any pandemic spread is the doubling time. It also measures the possibility of achieving flattened epidemic curve. Thus, it would be helpful if authors can present some result based on *doubling time*.

I believe that inclusion of these evidences will not only make the paper statistically stronger but also more conclusive.

**Other comments:**

This type of paper has tremendous scope of using various data visualization techniques to display the results. For example, authors should include more figures, charts, tables to display the changes related to the new case indices, absolute effect average, posterior probability and other indices. Similarly, wherever possible, try to use visualization tools instead of providing long elaboration. This will make the paper concise and visually appealing.
