# Supplementary material for: Early centralized isolation strategy for all confirmed cases of COVID-19 remains a core intervention to disrupt the pandemic spreading significantly
Source: PLoS One. 2021 Jul 15;16(7):e0254012. doi: 10.1371/journal.pone.0254012 (PMC8282022; doi:10.1371/journal.pone.0254012)
Supplement: Attachment — Submitted filename: Review_Report2.pdf [file pone.0254012.s013.pdf]

Manuscript title:

Early centralized isolation strategy for all confirmed cases of COVID-19 remains a core intervention to disrupt the pandemic spreading significantly

Manuscript Number: **PONE-D-20-33060R2**

Overall Remarks:

The revised version looks better now. The authors have included more charts/figures that make the paper visually more appealing. The analysis of doubling time and the reproduction rate have also been included. However, the work related to the reproduction rate (RR) lacks some important aspects. Authors need to improve that part.

Comments:

1. It is not clear which methodology was used for estimating the RR. The authors just mentioned that the RR was calculated by using the number of death cases. Exact name and relevant references are needed unless new method is proposed.

2. Before explaining empirical analysis, authors should explain the concept of RR and its importance (similarly for the doubling time analysis). Provide the formula of the corresponding metrics.

3. If possible, try to include figures of time-varying RR for all the relevant countries.
