# Supplementary material for: Early centralized isolation strategy for all confirmed cases of COVID-19 remains a core intervention to disrupt the pandemic spreading significantly
Source: PLoS One. 2021 Jul 15;16(7):e0254012. doi: 10.1371/journal.pone.0254012 (PMC8282022; doi:10.1371/journal.pone.0254012)
Supplement: Attachment — Submitted filename: Response to Reviewers.docx [file pone.0254012.s011.docx]

February 18^th^, 2021

PLOS ONE PONE-D-20-33060
**Early centralized isolation strategy for all confirmed cases of COVID-19 remains a core intervention to disrupt the pandemic spreading significantly**

Dear Editors and Reviewers,

We are most grateful to the Editor, the Associate Editor, and the Reviewer for providing helpful and constructive comments regarding our manuscript. We have taken all of these comments into account and hereby submit a revised manuscript with the changes underlined.

We responded to all of the comments as indicated below, and we hope that our explanations and revisions will be deemed satisfactory.

Sincerely,

Associate Nguyen Tien Huy, M.D, Ph.D.

School of Tropical Medicine and Global Health, Nagasaki University, Nagasaki 852-8523, Japan

E-mail: [tienhuy@nagasaki-u.ac.jp](mailto:tienhuy@nagasaki-u.ac.jp)

The comments of Academic Editor’s Siew Ann Cheong to the Author:

After careful consideration, we feel that it has merit but does not fully meet PLOS ONE’s publication criteria as it currently stands. Therefore, we invite you to submit a revised version of the manuscript that addresses the points raised during the review process.
In particular, please pay attention to the following comments, which are in my opinion the most important:

**Comment 1)** While both reviewers are happy that 13 countries and regions are compared in the manuscript, they also request that you explain why these 13 countries are selected, instead of others.

Author Response: Dear Editor, we are thankful for your comment, and we are pleased to answer your question. From 70 days from January, 22^nd^ 2020 (when the John Hopkins Coronavirus Resource Center began to collect the data of COVID-19) to the end of March 2020, we have collected data of countries with the highest number of daily confirmed cases. Among them, United States, Canada, and China, with the highest populations, were chosen due to their representative characteristic. Afterward, 5 Asian countries and 5 European countries with the percentage of days having the highest number of daily confirmed cases higher than 60% were chosen for later analysis. Additionally, Sweden, a North European country that decided to live in peace with the virus, was chosen as a European control country without both lockdown and centralized isolation. Details of the percentage of days having the highest number of daily confirmed cases of 13 countries were outlined in Supplementary table 1

*Text Insertion (if applicable)/ page/ line number of change:*

*(Page 4; line 100-109)*

“In order to assess the impact of current control measures on the number of new cases, during 70 days from 22 January 2020 (when John Hopkins Coronavirus Resource Center began to collect the data of COVID-19) to the end of March 2020, we have collected data of countries that had the highest number of daily confirmed cases. The United States, Canada, and China, with the highest population, were chosen due to their representative characteristic. Afterward, 5 Asian countries and 5 European countries with the percentage of days having the highest number of daily confirmed cases higher than 60% were chosen for later analysis. Additionally, Sweden, a North European country that decided to live in peace with the virus, was chosen as a European control country without both lockdown and centralized isolation. Details of the percentage of days having the highest number of daily confirmed cases of 13 countries were outlined in the S1 Table.”

S1 Table: 13 countries with the highest percentage of days having the highest number of daily confirmed cases

| **ID** | **Country** | **N (days)** | **Percentage of days having the highest number of daily confirmed cases** |
| --- | --- | --- | --- |
| **1** | China | 70 | 100.00% |
| **2** | South Korea | 70 | 100.00% |
| **3** | United State | 70 | 100.00% |
| **4** | France | 68 | 97.14% |
| **5** | Germany | 65 | 92.86% |
| **6** | Italy | 61 | 87.14% |
| **7** | United Kingdom | 61 | 87.14% |
| **8** | Japan | 60 | 85.71% |
| **9** | Canada | 53 | 75.71% |
| **10** | Singapore | 51 | 72.86% |
| **11** | Hong Kong | 48 | 68.57% |
| **12** | Spain | 47 | 67.14% |
| **13** | Taiwan | 43 | 61.43% |

**Comment 2)** The authors should provide references (like government announcements) that describe the control measures in greater detail.

Author Response: Dear Editor, thank you so much for your comment. An additional paragraph and supplementary table 5 (attached excel file) had been added to outline the detail of each control measures as below:

*Text Insertion (if applicable)/ page/ line number of change:*

*(Page 5-6; line 135-159)*

“In China, Taiwan, and Hong Kong, all suspected and confirmed cases were isolated in the healthcare facilities and could be ruled out after a negative of SARS-CoV-2 tests with at least 24-hour intervals regardless of the days of isolation. In South Korea and Singapore, a mandatory of 14 days of isolation was applied for all suspected and confirmed cases of COVID-19. In Japan, only suspected and confirmed cases with related symptoms were isolated at the hospitals, whereas the remaining cases without any symptoms were strictly quarantined at home. Even though the policy of centralized isolation of all confirmed cases was modified across these six countries, we assumed these control measures were comparable applied. Thus, centralized isolation of all confirmed cases is defined as a quarantine of all confirmed cases in hospitals, medical camps, or hotels under the supervision of medical staff and during a required time. Closure of schools was applied with the suspension of all cultural, educational, sports, and teaching activities. Closure of public areas consisted of non-essential business closures such as stores, shopping centers, services, such as bars, restaurants, cinemas, theaters, and prohibition of mass gathering. Additionally, citizens were requested not to leave the house unless for essential activities, always keep a safe distance (for example, at least 6-feet) and work from home. Closure of cities involved the restrictions on travel with the suspension of all forms of transportation across cities. Closure of borders is defined as the official announcement of the governments regarding the prohibition of entry and exit of all individuals from all entries, including lands, seas, rails, and air routes. Particularly, to address the heterogeneity of the date of implementation of control measures in countries with various states and cities such as the United States, Canada, and China, we assumed that the first date of applying the lockdown strategies is the representative date for these countries. Details of the date of the control measures mentioned above in each city and state of the United States, Canada, and China with reliable references were summarized in S5 table.”

**Comment 3)** Reviewer #2 also suggested a rough classification of the 13 countries selected into three categories: (a) Asian countries with strict lockdowns, (b) Asian countries without strict lockdowns, and (c) European and North American countries with strict lockdowns, and also using Sweden as an European country without lockdowns as a control.

Author Response: Dear Editor, thank you so much for your comment. Using Sweden as a control country have helped to reinforce and demonstrate the importance of the implementation of the five control measures such as (a) centralized isolation of all confirmed cases, (b) closure of schools, (c) closure of public areas, (d) closure of cities, and (e) closure of borders. Our analysis showed that when other countries began to start the preventive methods, Sweden, without any implementation of control measures, continued to increase the number of new cases. While Spain, Italy, the United Kingdom, Germany, and France lessened the day of achieving the highest new cases (day 29th, day 30th, day 47th, day 55th, and day 65th, respectively), Sweden reached the peak of cases in day 118^th^ with 62,728 confirmed cases according to government reports and 560,895 estimates. Additional analysis, writing, and update of figure 1 were added according to the recommendations and comments of the Reviewer as below:

*Text Insertion (if applicable)/ page/ line number of change:*

*(Page 4; line 91-94)*

“Thus, our study made an effort to summarize and highlight distinctive features of significant control measures among 14 particular countries, including China, Hong Kong, Taiwan, Singapore, Korea, Japan, the United States, France, Germany, the United Kingdom, Canada, Italy, Spain, and Sweden.”

*(Page 4; line 106-108)*

“Additionally, Sweden, a North European country that decided to live in peace with the virus, was chosen as a European control country without both lockdown and centralized isolation.”

*(Page 4-5; line 115-124)*

“Regarding Sweden, since the testing policy primarily focused on citizens with symptoms associating with COVID-19 infection and requirement of the inpatient hospital care and (or) elderly care, whereas people with mild symptoms were skipped for contacting the healthcare[3], the number of new confirmed cases of Sweden reported from JHU might be lower than the actual new confirmed cases in reality. To address this limitation, we have estimated the number of new confirmed cases in Sweden by using the number of new confirmed cases of neighboured European countries such as Spain, Italy, the United Kingdom, France, and Germany at the moment without application of any control measures. Our analysis revealed that the actual new confirmed cases of Sweden, in reality, was 8.47 times higher than the report of JHU (S2 Table).”

*(Page 8; line 234-235)*

“Sweden was excluded from all of the above control measures since this country denied to against the virus (Table 1).”

*(Page 13; line 265-270)*

“Based on the implementation of methods^a,b,c,d,e^, this study categorized 14 countries into two groups, group A and group B (Figure 1). Group A included Asian countries with strict lockdown policies (Taiwan, Hong Kong, Singapore, Japan, and China) and Asian countries without strict lockdowns (South Korea); they applied method^a^. Group B consisted of European and North American countries with strict lockdown policies (Canada, France, Germany, Spain, Italy, United Kingdom, and the United States) and Sweden; they did not apply method^a^”

*(Page 13, lune 286-287)*

*Figure 1:* Analysis of anti-epidemic solutions according to the number of COVID-19 case trajectories.

*(Page 14; line 304-310)*

As for Sweden, the number of estimated total confirmed cases (dash line) was quite comparable to that of countries in group B when the number of new cases has not reached its peak. In which Spain, Italy, the United Kingdom, Germany, and France lessened the day of achieving the highest new cases (day 29^th^, day 30^th^, day 47^th^, day 55^th^, and day 65^th^, respectively), Sweden reached the peak of cases in day 118^th^ with 62,728 confirmed cases according to government reports and 560,895 estimates.. The results show that when Sweden did not apply any measures^a,b,c,d,e^ and the peak time was 1.8 – 4.1 times longer than other countries.

*(Page 21, line 519-520)*

S2 Table: The average value of the ratio of daily new cases between the 5 countries and Sweden.

**Comment 4)** How the effectiveness of lockdowns would be affected by the delay between the time of infection and isolation. For your consideration, Reviewer #2 broke down this delay into three components: (a) the time between infection and onset of symptoms, (b) the onset of symptoms and the time of testing, and (c) the time of testing and reporting in official statistics.

Author Response: Dear Editor, we are thankful for your notice and completely agree with the declaration that the assumed period of 13 days might be a senseful approximation, and this delay consists of (at least) three time periods: A) time between infection and onset of symptoms (if any; incubation period), B) time between the onset of symptoms (if any) and testing, C) time between testing and reporting in official statistics. To solve this issue, we conducted the study with the hypothesis that the time between testing and reporting in official statistics could be fluctuating from one day to four days with the details were described in supplementary table 6. After the analysis, the results showed that the data changes due to the fluctuation of time between testing and reporting in official statistics led to insignificant differences of 0-3.5% (CI 95%). Thus, we decided to select day 13^th^ as a time point for analysis. An additional paragraph in the method section and in the limitation section of the discussion as well as supplementary table 6 have been added to clarify this matter.

*Text Insertion (if applicable)/ page/ line number of change:*

*(Page 7; line 194-207)*

“From the literature, the average incubation period for SARS-Cov-2 is 5.2 days with the 95% confidence interval of the distribution at 13 days^8^. However, we encountered a delay in the time reporting of data compared to the testing time in reality. There were some possible reasons for this. Firstly, there were 13 days of average incubation period as mentioned above. Secondly, the differences in time zones between 14 countries could lead to the differences in reporting the data to the public between JHU and these countries. Besides, from April 2020, the JHU database only updated data at the constant time from 3:30-4:00 pm (UTC). Thirdly, there was a lack of simultaneousness in reporting confirmed cases, recovered cases, and death cases of COVID-19. To solve this issue, we conducted the study with the hypothesis that the time between testing and reporting in official statistics could be fluctuating from one day to four days with the details were described in S6 table. After the analysis, the results showed that the data changes due to the fluctuation of time between testing and reporting in official statistics led to insignificant differences of 0-3.5% (CI 95%). Thus, we decided to select day 13^th^ as a time point for analysis.”

*(Page 18; line 431-433)*

Despite careful preparation, we acknowledged some limitations in our study that need to be identified. At first, the assumed period of 14 days might be a senseful approximation since the time between the onset of symptoms and testing is hardly detectable.

*(Page 21, line 525-526)*

**S6 Table: Insignificant data changes due to the fluctuation of time between testing and reporting in official statistics**

**Comment 5)** Reviewer #1 would like to see more discussions on the limitations of this study, so as to guide future studies.

Author Response: Dear Editor, thank you so much for your comment. As your suggestion, we have updated the limitation sections as below:

*Text Insertion (if applicable)/ page/ line number of change:*

*(Page 18; line 431-447)*

“Despite careful preparation, we acknowledged some limitations in our study that need to be identified. At first, the assumed period of 14 days might be a senseful approximation since the time between the onset of symptoms and testing is hardly detectable. Even though our study demonstrated the crucial role of centralized isolation in fighting the pandemic and may make other measures superfluous, but only if this measure is imposed at an early stage of virus spread, we also face a current challenge of identification all infections at an early stage of the outbreak. Detection of asymptomatic but infected people who had never been tested represented as an important matter that needed to be discussed. Besides, Asian countries with smaller territory sizes and accumulated gaining experiences of SAR-CoV-1 in the past had a greater benefit to tackle the spread of this infectious disease than European and American countries. Furthermore, Asian countries with “authoritarian government” and “collectivist culture” that pushed societal interests to the forefront might be easily adapted the lockdown policy. In contrast, European and American countries where “individualistic culture” is dominant are challenging to comply with strict enforcement of regulations. Finally, the heterogeneity in the analysis is unavoidable due to difficulties to reach a comparable definition of the time for each measure in countries such as the United States, Canada, and China. Also, there were differences in testing policies and the criteria of confirmes case in each country.”

**Comment 6)** Please include the following items when submitting your revised manuscript:

- A rebuttal letter that responds to each point raised by the academic editor and reviewer(s). You should upload this letter as a separate file labeled 'Response to Reviewers'.
- A marked-up copy of your manuscript that highlights changes made to the original version. You should upload this as a separate file labeled 'Revised Manuscript with Track Changes'.
- An unmarked version of your revised paper without tracked changes. You should upload this as a separate file labeled 'Manuscript'.

Author Response: Dear Editor, we have included all of these three required items in our submission. Thank you so much for your announcement.

**Comment 7)** When submitting your revision, we need you to address these additional requirements.

1. Please ensure that your manuscript meets PLOS ONE's style requirements, including those for file naming. The PLOS ONE style templates can be found at

https://journals.plos.org/plosone/s/file?id=wjVg/PLOSOne_formatting_sample_main_body.pdf and

<https://journals.plos.org/plosone/s/file?id=ba62/PLOSOne_formatting_sample_title_authors_affiliations.pdf>

2. Please ensure links to all stated data sources have been included or cited in the methods section.

3. Thank you for stating the following financial disclosure:

"The funders had no role in study design, data collection and analysis, decision to publish, or preparation of the manuscript,"

Author Response: Dear Editor, thank you so much for your announcement. We have addressed all of the above issues in our revised manuscript.

**Comment 8)** At this time, please address the following queries:

a. Please clarify the sources of funding (financial or material support) for your study. List the grants or organizations that supported your study, including funding received from your institution.
b. State what role the funders took in the study. If the funders had no role in your study, please state: “The funders had no role in study design, data collection and analysis, decision to publish, or preparation of the manuscript.”
c. If any authors received a salary from any of your funders, please state which authors and which funders.
d. If you did not receive any funding for this study, please state: “The authors received no specific funding for this work.”

Please include your amended statements within your cover letter; we will change the online submission form on your behalf.

Author Response: Dear Editor, thank you so much for your comment. We have addressed all of the above issues in our revised manuscript.

**Reviewer #1:** The article addresses a relevant and current topic. Explains the scientific background and rationale of the investigation. The methods section describes the setting, locations and relevant dates. The criteria of selection of countries were described.

**Comment 1)** However, some elements could be better described. Data from the World Health Organization (March, 15) demonstrated that other countries have more cases or mortality, especially in Europe. Thus, it is important to explain why these countries were not selected.

Author Response: Dear Reviewer, we are thankful for your comment, and we are pleased to answer your question. From 70 days from January, 22^nd^ 2020 (when the John Hopkins Coronavirus Resource Center began to collect the data of COVID-19) to the end of March 2020, we have collected data of countries with the highest number of daily confirmed cases. Among them, United States, Canada, and China, with the highest populations, were chosen due to their representative characteristic. Afterward, 5 Asian countries and 5 European countries with the percentage of days having the highest number of daily confirmed cases higher than 60% were chosen for later analysis. Additionally, Sweden, a North European country that decided to live in peace with the virus, was chosen as a European control country without both lockdown and centralized isolation. Details of the percentage of days having the highest number of daily confirmed cases of 13 countries were outlined in supplementary table 1.

*Text Insertion (if applicable)/ page/ line number of change:*

*(Page 4; line 100-109)*

“In order to assess the impact of current control measures on the number of new cases, during 70 days from 22 January 2020 (when the John Hopkins Coronavirus Resource Center began to collect the data of COVID-19) to the end of March 2020, we have collected data of countries that had the highest number of daily confirmed cases. The United States, Canada, and China, with the highest population, were chosen due to their representative characteristic. Afterward, 5 Asian countries and 5 European countries with the percentage of days having the highest number of daily confirmed cases higher than 60% were chosen for later analysis. Additionally, Sweden, a North European country that decided to live in peace with the virus, was chosen as a European control country without both lockdown and centralized isolation. Details of the percentage of days having the highest number of daily confirmed cases of 13 countries were outlined in the S1 Table.”

*(Page 21, line 517-518)*

S1 Table: 13 countries with the highest percentage of days having the highest number of daily confirmed cases

| **ID** | **Country** | **N (days)** | **Percentage of days having the highest number of daily confirmed cases** |
| --- | --- | --- | --- |
| **1** | China | 70 | 100.00% |
| **2** | South Korea | 70 | 100.00% |
| **3** | United State | 70 | 100.00% |
| **4** | France | 68 | 97.14% |
| **5** | Germany | 65 | 92.86% |
| **6** | Italy | 61 | 87.14% |
| **7** | United Kingdom | 61 | 87.14% |
| **8** | Japan | 60 | 85.71% |
| **9** | Canada | 53 | 75.71% |
| **10** | Singapore | 51 | 72.86% |
| **11** | Hong Kong | 48 | 68.57% |
| **12** | Spain | 47 | 67.14% |
| **13** | Taiwan | 43 | 61.43% |

**Comment 2)** Data relative to control measures could be better described.

Author Response: Dear Reviewer, we are thankful for your comments. An additional paragraph and supplementary table 5 (attached excel file) had been added to outline the detail of each control measures as below:

*Text Insertion (if applicable)/ page/ line number of change:*

*(Page 5-6; line 135-159)*

***“***In China, Taiwan, and Hong Kong, all suspected and confirmed cases were isolated in the healthcare facilities and could be ruled out after a negative of SARS-CoV-2 tests with at least 24-hour intervals regardless of the days of isolation. In South Korea and Singapore, a mandatory of 14 days of isolation was applied for all suspected and confirmed cases of COVID-19. In Japan, only suspected and confirmed cases with related symptoms were isolated at the hospitals, whereas the remaining cases without any symptoms were strictly quarantined at medical camps, or hotels under medical staff supervision and during a required time. Even though the policy of centralized isolation of all confirmed cases was modified across these six countries, we assumed these control measures were comparable applied. Thus, centralized isolation of all confirmed cases is defined as a quarantine of all confirmed cases in hospitals, medical camps, or hotels under the supervision of medical staff and during a required time. Closure of schools was applied with the suspension of all cultural, educational, sports, and teaching activities. Closure of public areas consisted of non-essential business closures such as stores, shopping centers, services, such as bars, restaurants, cinemas, theaters, and prohibition of mass gathering. Additionally, citizens were requested not to leave the house unless for essential activities, always keep a safe distance (for example, at least 6-feet) and work from home. Closure of cities involved the restrictions on travel with the suspension of all forms of transportation across cities. Closure of borders is defined as the official announcement of the governments regarding the prohibition of entry and exit of all individuals from all entries, including lands, seas, rails, and air routes. Particularly, to address the heterogeneity of the date of implementation of control measures in countries with various states and cities such as the United States, Canada, and China, we assumed that the first date of applying the lock-down strategies is the representative date for these countries. Details of the date of the control measures mentioned above in each city and state of the United States, Canada, and China with reliable references were summarized in S5 table.”

**Comment 3)** The documents used as a reference to identify control measures must be referenced in the text for readers to consult. It is necessary to revise these references. Data from Spain (table 1) for example point to the closure of schools on March 11. However, this only occurred in the entire country after March 14. The same situation applies to control relaxation measures. In Spain, the control reduction plan is published on April 28, 2020 with different phases in each autonomous community.

Author Response: Dear Reviewer, we are thankful for your kind detections. To address the heterogeneity of the date of implementation of control measures in countries with various states and cities, such as the United States, Canada, Spain, and China, we assumed that the first date of applying the lock-down strategies is the representative date for these countries. For example, with the closure of schools in Spain, we chose the first date that Spain used this measure in Madrid on 11^th^ March 2020 (reference as below). As your suggestions, we have adjusted the data in table 1 and we have also added an Supplementary Table 7 with the link of reliable references for readers to consult. Additionally, we also re-analyzed our data, and the new findings are not modified.

[https://edition.cnn.com/world/live-news/coronavirus-outbreak-03-11-20-intl-hnk/h_00ffe686205aac4779223e09fb9bbd0e](https://edition.cnn.com/world/live-news/coronavirus-outbreak-03-11-20-intl-hnk/h_00ffe686205aac4779223e09fb9bbd0e?fbclid=IwAR2apxkIHjdtAEcjUnh90pfKZvDYPz56rzGXHPMWdDI0IaKx84nbgKOM9Io)

**Comment 4)** Another aspect in relation to control measures is the difficulty of establishing a date and intensity of measures in continental countries such as China, Canada and the United States. I highlight the situation in the USA, where each state institutes different measures. This question should be clearer in the methods, results tables and limitations of the Study.

Author Response: Dear Reviewer, we are thankful for your comment and totally agree with this remark. To address the heterogeneity of the date of implementation of control measures in countries with various states and cities such as the United States, Canada, and China, we assumed that the first date of application of the lock-down strategies is the representative date for these countries. We have acknowledged this limitation and mentioned it in the limitation section of this manuscript. An additional paragraph had been added to clarify this matter.

*Text Insertion (if applicable)/ page/ line number of change:*

*(Page 6; line 154-159)*

“Particularly, to address the heterogeneity of the date of implementation of control measures in countries with various states and cities, such as the United States, Canada, Spain, and China, we assumed that the first date of applying the lock-down strategies is the representative date for these countries. Details of the date of the control measures mentioned above in each city and state of the United States, Canada, Spain, and China with reliable references were summarized in S5 table.”

*(Page 18 ; line 444-447)*

“Finally, the heterogeneity in the analysis is unavoidable due to difficulties to reach a comparable definition of the time for each measure in countries such as the United States, Canada, and China. Also, there were differences in testing policies and the criteria of confirmes case in each country.”

**Comment 5)** Another relevant aspect is that it would be important to describe what was considered centralized isolation of all confirmed cases in the method section. Guidelines recommend isolation for all cases in most countries (including the WHO guideline). Thus, it is important for reads understand how the researchers define the difference between countries and references documents (the isolations were compulsory?). Other factors to understand the control measures like testing strategies were not described, analysed in the method section or mentioned in the discussion section. This aspect is important because a country could define isolation of all cases and do not test their citizens adequately, for example, and do not isolate a great part of the infected citizens. The statistics analysis must be described better in a supplementary document or in the text.

Author Response: Dear Reviewer, we totally agree with your remark regarding the modification of centralization of all confirmed cases according to each country. In China, Taiwan, and Hong Kong, all suspected and confirmed cases were isolated in the healthcare facilities and could be ruled out after a negative of SARS-CoV-2 tests with at least 24-hour intervals regardless of the days of isolation. In South Korea and Singapore, a mandatory of 14 days of isolation was applied for all suspected and confirmed cases of COVID-19. In Japan, only suspected and confirmed cases with related symptoms were isolated at the hospitals, whereas the remaining cases without any symptoms were strictly quarantined at medical camps, or hotels under medical staff supervision and during a required time. Even though the policy of centralized isolation of all confirmed cases was modified across these six countries, we assumed these control measures were comparable applied. Thus, centralized isolation of all confirmed cases is defined as a quarantine of all confirmed cases in hospitals, medical camps, or hotels under the supervision of medical staff and during a required time. An additional paragraph had been added to clarify this issue as below:

*Text Insertion (if applicable)/ page/ line number of change:*

*(Page 5; line 135-146)*

In China, Taiwan, and Hong Kong, all suspected and confirmed cases were isolated in the healthcare facilities and could be ruled out after a negative of SARS-CoV-2 tests with at least 24-hour intervals regardless of the days of isolation. In South Korea and Singapore, a mandatory of 14 days of isolation was applied for all suspected and confirmed cases of COVID-19. In Japan, only suspected and confirmed cases with related symptoms were isolated at the hospitals, whereas the remaining cases without any symptoms were strictly quarantined at medical camps, or hotels under medical staff supervision and during a required time. Even though the policy of centralized isolation of all confirmed cases was modified across these six countries, we assumed these control measures were comparable applied. Thus, centralized isolation of all confirmed cases is defined as a quarantine of all confirmed cases in hospitals, medical camps, or hotels under the supervision of medical staff and during a required time.

**Comment 6)** The results were well described, however it could be necessary to correct some results (changes in the definition of date of implementation of the national intervention and analysis of other countries).

Author Response: Dear Reviewer, we are thankful for your comment and totally agree with this remark. After modification of dates of implementation of the national interventions, updated analysis results and updated figures had been added to address these modifications.

*Text Insertion (if applicable)/ page/ line number of change:*

*(Page 9; line 238-239)*

Table 1: Date of implementation of the national intervention at the moment of the 10th confirmed cases

*(Page 11; line 263)*

Table 2. Decreasing new case indices of countries by intervention solution

*(Page 13; line 286-287)*

Figure 1: Analysis of anti-epidemic solutions according to the number of COVID-19 cases trajectories.

**Comment 7)** The discussion section could explore other hypothesis and aspects that could justify differences. All countries in group A were European or Americans with occidental culture. The group B has only Asiatic regions/countries. The experience with SAR-COV 1 was not mentioned like one aspect of difference between Asiatic and European and American Countries results. Different territory sizes and governance differences were not mentioned too (Asiatic regions were smaller and some are islands - these are importants factors in epidemic control). It could be important to describe the differences.

Author Response: Dear Reviewer, we totally agree with your comprehensive comments. Based on your suggestions, we made an update regarding these matters in the discussion section as below:

*Text Insertion (if applicable)/ page/ line number of change:*

*(Page 18; line 438-444)*

Besides, Asian countries with smaller territory sizes and accumulated gaining experiences of SAR-CoV-1 in the past had a greater benefit to tackle the spread of this infectious disease than European and American countries. Furthermore, Asian countries with “authoritarian government” and “collectivist culture” that pushed societal interests to the forefront might be easily adapted the lockdown policy. In contrast, European and American countries where “individualistic culture” is dominant are challenging to comply with strict enforcement of regulations.

**Comment 8)** The study limitations could be better described (difficulties to defines the time of each measure, cases criteria definitions in which country, testing policies, governance differences etc). The conclusions were well described and were adequate to the results.

Author Response: Dear Reviewer, we are thankful for your remark. Based on your corrections, we have updated the limitation sections as below:

*Text Insertion (if applicable)/ page/ line number of change:*

*(Page 18; line 431-447)*

“Despite careful preparation, we acknowledged some limitations in our study that need to be identified. At first, the assumed period of 14 days might be a senseful approximation since the time between the onset of symptoms and testing is hardly detectable. Even though our study demonstrated the crucial role of centralized isolation in fighting the pandemic and may make other measures superfluous, but only if this measure is imposed at an early stage of virus spread, we also face a current challenge of identification all infections at an early stage of the outbreak. Detection of asymptomatic but infected people who had never been tested represented as an important matter that needed to be discussed. Besides, Asian countries with smaller territory sizes and accumulated gaining experiences of SAR-CoV-1 in the past had a greater benefit to tackle the spread of this infectious disease than European and American countries. Furthermore, Asian countries with “authoritarian government” and “collectivist culture” that pushed societal interests to the forefront might be easily adapted the lockdown policy. In contrast, European and American countries where “individualistic culture” is dominant are challenging to comply with strict enforcement of regulations. Finally, the heterogeneity in the analysis is unavoidable due to difficulties to reach a comparable definition of the time for each measure in countries such as the United States, Canada, and China. Also, there were differences in testing policies and the criteria of confirmes case in each country.”

**Reviewer #2:** The authors attempt to assess the effectiveness of nonpharmaceutical interventions in fighting the SARS-CoV-2 pandemic. The study incorporates 13 countries and compares their strategies. From the methodological point of view, infections are analyzed over time, whilst testing the influence of different measures on the temporal development of infections. The authors conclude that centralized isolation of confirmed infected individuals is an effective strategy (on condition that is implemented early) rather than “lockdowns”. The study is very interesting and of high quality from a methodological perspective. The simultaneous comparison of different countries (with different economic and cultural background) is to be welcomed. However, there are some issues I would recommend to adress in a revision (“major revisions”), especially with respect to the selection of countries and the interpretation of the results (see below).

MAJOR COMMENTS
**Comment 1)** Again, I have to express my happiness about the comparison of different countries with different containment strategies. However, there is an important issue regarding the selection of these countries: With respect to the “lockdown” policy, the selected countries in the study can be divided into three groups (not to be confused with the classification made by the authors on page 8): A) Asian countries WITH strict “lockdowns”, B) Asian countries WITHOUT strict “lockdowns” and C) European and North American countries WITH strict “lockdowns”. However, there is one North European country (Sweden) which did NOT impose a “lockdown” as well (Note: We have to keep in mind that there have been several measures against SARS-CoV-2 spread both mandatory and voluntary – it is a common “fairy tale” that Sweden did nothing against the virus! Furthermore, assessing the Sweden strategy is complicated as, in the first wave of infections, Sweden had a much higher COVID mortality than its neighbours but a lower mortality compared to France, Italy, or Belgium with hard “lockdown” measures). But I would strongly recommend including Sweden into the analysis as a control group (European country WITHOUT “lockdown” AND without centralized isolation).
See this paper on Sweden by Born et al. 2020: `.

Author Response: Dear Reviewer, we are thankful for your valuable suggestion, and we totally agree with this remark. Using Sweden as a control country have helped to reinforce and demonstrate the importance of the implementation of the five control measures such as (a) centralized isolation of all confirmed cases, (b) closure of schools, (c) closure of public areas, (d) closure of cities, and (e) closure of borders. Our analysis showed that when other countries began to start the preventive methods, Sweden, without any implementation of control measures, continued to increase the number of new cases. While Spain, Italy, the United Kingdom, Germany, and France lessened the day of achieving the highest new cases (day 29th, day 30th, day 47th, day 55th, and day 65th, respectively), Sweden reached the peak of cases in day 118th with 62,728 confirmed cases according to government reports and 560,895 estimates. Additional analysis, writing, and update of figure 1 were added according to the recommendations and comments of the Reviewer as below:

*Text Insertion (if applicable)/ page/ line number of change:*

*(Page 4; line 91-94)*

“Thus, our study made an effort to summarize and highlight distinctive features of significant control measures among 14 particular countries, including China, Hong Kong, Taiwan, Singapore, Korea, Japan, the United States, France, Germany, the United Kingdom, Canada, Italy, Spain, and Sweden.”

*(Page 4; line 106-108)*

“Additionally, Sweden, a North European country that decided to live in peace with the virus, was chosen as a European control country without both lockdown and centralized isolation.”

*(Page 4-5; line 115-124)*

“Regarding Sweden, since the testing policy primarily focused on citizens with symptoms associating with COVID-19 infection and requirement of the inpatient hospital care and (or) elderly care, whereas people with mild symptoms were skipped for contacting the healthcare[3], the number of new confirmed cases of Sweden reported from JHU might be lower than the actual new confirmed cases in reality. To address this limitation, we have estimated the number of new confirmed cases in Sweden by using the number of new confirmed cases of neighboured European countries such as Spain, Italy, the United Kingdom, France, and Germany at the moment without application of any control measures. Our analysis revealed that the actual new confirmed cases of Sweden, in reality, was 8.47 times higher than the report of JHU (S2 Table).”

*(Page 8; line 234-235)*

“Sweden was excluded from all of the above control measures since this country denied to against the virus (Table 1).”

*(Page 13; line 265-270)*

“Based on the implementation of methods^a,b,c,d,e^, this study categorized 14 countries into two groups, group A and group B (Figure 1). Group A included Asian countries with strict lockdown policies (Taiwan, Hong Kong, Singapore, Japan, and China) and Asian countries without strict lockdowns (South Korea); they applied method^a^. Group B consisted of European and North American countries with strict lockdown policies (Canada, France, Germany, Spain, Italy, United Kingdom, and the United States) and Sweden; they did not apply method^a^”

*(Page 13, line 286-287)*

*Figure 1:* Analysis of anti-epidemic solutions according to the number of COVID-19 case trajectories.

*(Page 14; line 304-310)*

As for Sweden, the number of estimated total confirmed cases (dash line) was quite comparable to that of countries in group B when the number of new cases has not reached its peak. In which Spain, Italy, the United Kingdom, Germany, and France lessened the day of achieving the highest new cases (day 29^th^, day 30^th^, day 47^th^, day 55^th^, and day 65^th^, respectively), Sweden reached the peak of cases in day 118^th^ with 62,728 confirmed cases according to government reports and 560,895 estimates. The results show that when Sweden did not apply any measures^a,b,c,d,e^ and the peak time was 1.8 – 4.1 times longer than other countries.

*(Page 20)*

S2 Table: The average value of the ratio of daily new case between the 5 countries and Sweden.

**Comment 2)** As the analysis incorporates infection/fatalities data for 13 countries, it is reasonable to use a data source which provides all this case data for each country. Like in many studies towards the effectiveness of nonpharmaceutical interventions, the database from the Johns Hopkins University (JHU) was used. This data does, of course, not contain information about the true infection date (which is unknown in the majority of cases). By consequence, valid analyses of the effectiveness of nonpharmaceutical interventions must incorporate an appropriate delay between infection an the (possible) impact of measures. In the present study, the authors consider this problem by referring to the 95% percentile of the SARS-CoV-2/COVID-19 incubation period from the literature. The assumed period of 13 days might be a senseful approximation. However, this delay consists of (at least) three time periods: A) time between infection and onset of symptoms (if any; incubation period), B) time between onset of symptoms (if any) and testing, C) time between testing and reporting in official statistics (including JHU database). Note that these time periods may overlap. The authors should clarify this and should comment how this assumption on the delay might affect the results.
For further information on the issue of reporting delays etc., see for example the following literature:
Flaxman et al 2020 (https://doi.org/10.1038/s41586-020-2405-7) and Homburg 2020 (https://doi.org/10.1515/ev-2020-0010) also use JHU data for international comparisons of NPI effectiveness, but they trace infection dates back from reported death cases. A similar method is used by Wood 2020 (https://arxiv.org/pdf/2005.02090.pdf). A critique on former NPI studies using the JHU data (explicitly discussing the “reporting delay problem”) can be found in Wieland 2020 (https://doi.org/10.1016/j.ssci.2020.104924).

Author Response: Dear Reviewer, we are thankful for your kind comment and completely agree with the declaration that the assumed period of 13 days might be a senseful approximation, and this delay consists of (at least) three time periods: A) time between infection and onset of symptoms (if any; incubation period), B) time between the onset of symptoms (if any) and testing, C) time between testing and reporting in official statistics. To solve this issue, we conducted the study with the hypothesis that the time between testing and reporting in official statistics could be fluctuating from one day to four days with the details were described in supplementary table 6. After the analysis, the results showed that the data changes due to the fluctuation of time between testing and reporting in official statistics led to insignificant differences of 0-3.3% (CI 95%). Thus, we decided to select day 13^th^ as a time point for analysis. An additional paragraph in the method section and in the limitation section of the discussion as well as supplementary table 6 have been added to clarify this matter.

*Text Insertion (if applicable)/ page/ line number of change:*

*(Page 7; line 194-207)*

“From the literature, the average incubation period for SARS-Cov-2 is 5.2 days with the 95% confidence interval of the distribution at 13 days^8^. However, we encountered a delay in the time reporting of data compared to the testing time in reality. There were some possible reasons for this. Firstly, there were 13 days of average incubation period as mentioned above. Secondly, the differences in time zones between 14 countries could lead to the differences in reporting the data to the public between JHU and these countries. Besides, from April 2020, the JHU database only updated data at the constant time from 3:30-4:00 pm (UTC). Thirdly, there was a lack of simultaneousness in reporting confirmed cases, recovered cases, and death cases of COVID-19. To solve this issue, we conducted the study with the hypothesis that the time between testing and reporting in official statistics could be fluctuating from one day to four days with the details were described in S6 table. After the analysis, the results showed that the data changes due to differences in time zones between JHU and 14 countries led to insignificant differences of 0-3.3% (CI 95%). Thus, we decided to select day 13^th^ as a time point for analysis.“

*(Page 18; line 431-433)*

“Despite careful preparation, we acknowledged some limitations in our study that need to be identified. At first, the assumed period of 14 days might be a senseful approximation since the time between the onset of symptoms and testing is hardly detectable.”

*(Page 20)*

S6 Table: Insignificant data changes due to the fluctuation of time between testing and reporting in official statistics

**Comment 3)** There is an issue with respect to the interpretation of the effectiveness of CENTRALIZED isolation: “[…] is not sufficient when we let the confirmed cases be self-isolated at home. The rationale behind this is that these individuals continue to do their daily activities such as buying food, entering local pharmacies, and patronizing other businesses, which might result in an increase in the risk of community spread.” (page 11, lines 323-326). I cannot assess the practical implementation of isolation in Singapore or Taiwan but, in European countries, domestic quarantine means a strict stay-at-home order which is controlled by health authorities. Is the centralized isolation strategy efficient because it is CENTRALIZED or because other types of isolation (such as domestic quarantine) are not or cannot be controlled? This is a more general issue, as, in the “Discussion” section, measures are supported because of their rigour: “However, as human nature dictates, adherence to isolation and other strategies e.g social distancing and wearing masks, can be difficult when not strictly enforced. Thus, protocols for enhancing community compliance with the secondary measures to further contain the spread, must be studied” (page 13, lines 375-378). How does this conclusion match the results from several European countries with differing NPIs and differing levels of self-responsibility? See e.g point 1 with respect to Sweden. I would not agree that any measure should be on a volunteer basis, but, in democratic states under the rule of law, governments must count on the support of individuals. I would ask the authors to include these aspects in the discussion, because voluntary compliance to (reasonable) rules are an important foundation of democratic societies.

Author Response: Dear Reviewer, we totally agree with your remark regarding voluntary compliance to (reasonable) rules as a vital foundation of democratic societies. The effectiveness of centralized isolation of all confirmed cases in various countries in our study is based on if the governments supported their citizens to comply with the control measure. In our study, centralized isolation of all confirmed cases is defined as a quarantine of all confirmed cases in hospitals, medical camps, or hotels under medical staff supervision and during a required time. In China, Taiwan, and Hong Kong, all suspected and confirmed cases were isolated in the healthcare facilities and could be ruled out after a negative of SARS-CoV-2 tests with at least 24-hour intervals regardless of the days of isolation. In South Korea and Singapore, a mandatory of 14 days of isolation was applied for all suspected and confirmed cases of COVID-19. To further strengthen the measure's compliance, South Korea activated the criminal penalty on violating the isolation order on February 26^th^, 2020^1^. Besides isolating all suspected cases and confirmed cases at designated locations, Hong Kong also uniquely monitored people who quarantined at their homes by electrical wristbands^2^. In contrast, the USA, Canada, the UK, France, Germany, Italy, and Spain did not use centralized isolation of all confirmed cases. Furthermore, in European and American countries where “individualistic culture” is dominant, this is difficult to comply with strict enforcement of regulations. In contrast, Asian countries with “authoritarian government” and “collectivist culture” that pushed societal interests to the forefront might be easily adapted the lockdown policy.

^1^<http://www.korea.kr/common/download.do?fileId=190536078&tblKey=GMN>

^2^<https://www.news.gov.hk/eng/2020/02/20200203/20200203_150504_450.html?type=category&name=covid19&tl=t>

*Text Insertion (if applicable)/ page/ line number of change:*

*(Page 5; line 135-144)*

“In China, Taiwan, and Hong Kong, all suspected and confirmed cases were isolated in the healthcare facilities and could be ruled out after a negative of SARS-CoV-2 tests with at least 24-hour intervals regardless of the days of isolation. In South Korea and Singapore, a mandatory of 14 days of isolation was applied for all suspected and confirmed cases of COVID-19. In Japan, only suspected and confirmed cases with related symptoms were isolated at the hospitals, whereas the remaining cases without any symptoms were strictly quarantined were strictly quarantined at medical camps, or hotels under medical staff supervision and during a required time. Even though the policy of centralized isolation of all confirmed cases was modified across these six countries, we assumed these control measures were comparable applied.”

*(Page 17; line 440-444)*

“Furthermore, Asian countries with “authoritarian government” and “collectivist culture” that pushed societal interests to the forefront might be easily adapted the lockdown policy. In contrast, European and American countries where “individualistic culture” is dominant are challenging to comply with strict enforcement of regulations.”

MINOR COMMENTS

**Comment 4)** The criteria for county selection appear reasonable in most cases, but please describe (just 1-2 sentences) what is meant by “having a close relationship with the outbreak's origin (Wuhan, China)”.

Author Response: Dear Reviewer, after revising the manuscript, we decide to remove this sentence and insert a new paragraph to clarify the selection strategy of the targeted countries in our study as below:

*Text Insertion (if applicable)/ page/ line number of change:*

*(Page 4; line 100-109)*

***“***In order to assess the impact of current control measures on the number of new cases, during 70 days from 22 January 2020 (when John Hopkins Coronavirus Resource Center began to collect the data of COVID-19) to the end of March 2020, we have collected data of countries that had the highest number of daily confirmed cases. The United States, Canada, and China, with the highest population, were chosen due to their representative characteristic. Afterward, 5 Asian countries and 5 European countries with the percentage of days having the highest number of daily confirmed cases higher than 60% were chosen for later analysis. Additionally, Sweden, a North European country that decided to live in peace with the virus, was chosen as a European control country without both lockdown and centralized isolation. Details of the percentage of days having the highest number of daily confirmed cases of 13 countries were outlined in the S1 Table.”

*(Page 21; line 517-518)*

S1 Table: 13 countries with the highest percentage of days having the highest number of daily confirmed cases

**Comment 5)** Centralized isolation is identified as an effective strategy in fighting the pandemic and may make other measures superfluous, but only if this measure is imposed at an early stage of virus spread (“On the other hand, the application of centralized isolation would no longer be effective without other solutions such as closing public places, schools, cities, or borders; when the number of active cases was over 400”; page 9, lines 265-267). We know that SARS-CoV-2 cases are underestimated everywhere because of an unknown “dark figure” of asymptomatic but infected people who have never been tested. However, official reported cases are the only data source available, and, according the WHO, “asymptomatically infected individuals are much less likely to transmit the virus than those who develop symptoms” (http://www.emro.who.int/health-topics/corona-virus/transmission-of-covid-19-by-asymptomatic-cases.html). Is it realistic to identify all infections at an early stage of the pandemic? I would recommend that the authors should reflect this issue, just in 1-2 sentences.

Author Response: Dear Reviewer, your above comment was exact and comprehensive. It’s obvious that identify all infections at an early stage of the pandemic is challengeable and even irrealistic. We have addressed these ideas in the limitation section as below:

*Text Insertion (if applicable)/ page/ line number of change:*

*(Page 18; line 433-438)*

Even though our study demonstrated the crucial role of centralized isolation in fighting the pandemic and may make other measures superfluous, but only if this measure is imposed at an early stage of virus spread, we also face a current challenge of identification all infections at an early stage of the outbreak. Detection of asymptomatic but infected people who had never been tested represented as an important matter that needed to be discussed.

**Comment 6)** Page 6, line 156: “Since vaccines and target drugs are not available…” should be more like “Since vaccines and target drugs were not available in March 2020…”. A similar issue is on page 11, line 319: “…while vaccination and approved drugs for COVID-19 are still unavailable…”. Note that I received the review invitation on December 12, 2020, and I don’t know when the manuscript was written and submitted. Now, there is a vaccine.

Author Response: Dear Reviewer, we totally agree to your comments. An adjustment of these two sentences had been inserted as below:

*Text Insertion (if applicable)/ page/ line number of change:*

*(Page 6; line 171-172)*

“Since vaccines and target drugs were not available in March 2020, current interventions can only control the number of new cases.”

*(Page 16; line 368-370)*

“Currently, while vaccination and approved drugs for COVID-19 require time to assess its efficaciousness, the management of the sources of infection, as well as routes of transmission, remain the vital factors to control the pandemic.”

**Comment 7)** The authors should refer some previous literature towards analyses of the effectiveness of NPIs, especially country comparisons. See the literature in point 2 and, e.g., Chaudry et al 2020 (https://doi.org/10.1016/j.eclinm.2020.100464).

Author Response: Dear Reviewer, thank you so much for your kind recommendation. After reading this paper, we have added some sentences to refer to the previous findings towards the effectiveness of NPIs as below:

*Text Insertion (if applicable)/ page/ line number of change:*

*(Page 17; line 395-399)*

“In the same manner, our findings were in line with the report of Chaudhry et al., which identified that the days to partial or full lockdown and the day to any border closures were the two significant predictors associating with the total number of reported cases per million^14^. Interestingly, this author also figured out that a full lockdown policy was a supporting factor in increasing the number of recovered cases.”

**Comment 8)** On page 13, lines 385-386, the authors write that “lockdown” policies have “a negative impact on the society and economy”. Like the majority of scientists, I would agree with that. However, it may be a good idea to cite some literature on negative impacts of “lockdowns” already in the “Introduction” section.
For example, Miles et al. 2020 (https://doi.org/10.1111/ijcp.13674) balance the costs and benefits of “lockdowns” and find that the economic impacts are more harmful than their benefits (economic consequences). Adams-Prassl et al 2020 (https://hceconomics.uchicago.edu/research/working-paper/impact-coronavirus-lockdown-mental-health-evidence-us) identify severe impacts of stay-at-home orders on mental health (psychosocial consequences).

Author Response: Dear Reviewer, we are thankful for your kind comments with the two valuable suggested articles. Additional sentences have been inserted into our manuscript to mention the negative impacts of lockdown policy in the introduction section as below:

*Text Insertion (if applicable)/ page/ line number of change:*

*(Page 3-4; line 83-91)*

“However, these lockdown policies are hurting with negative impacts in multiple aspects. Miles et al. determined that the lockdown cost was 40% higher than the highest benefits from avoiding the worst mortality case scenario at full life expectancy tariff^1^. For mental health, Adams-Prassl et al. recognized that citizens living in states with implementation of lockdown scored 0.085 standard deviations lower on the standardized WHO-5 mental health index compared to those living in states without lockdown strategy^2^. Despite its negative impacts, potentially measuring implementation also effectively proved its ability to control this pandemic and remains a question that we hope to elucidate.”

**Comment 9)** Of course, it’s a matter of style but maybe the authors should use the term “nonpharmaceutical interventions” at least once in the text and/or the abstract (because this is the “de facto official” term for containment measures in the international literature). But, keep in mind that this is a “style question” :)

Author Response: Dear Reviewer, we agree with your suggestion regarding the use of the term “non-pharmaceutical interventions.” Adjustments had been inserted in the manuscript as below:

*Text Insertion (if applicable)/ page/ line number of change:*

*(Page 2; line 48-50)*

“However, when the number of cases increased with the apparition of new clusters, coordination between centralized isolation and non-pharmaceutical interventions would facilitate controlling the crisis efficiently.”

*(Page 3; line 80-81)*

“A list of non-pharmaceutical interventions at different levels and various strategies had been established to prevent and mitigate the continual spread of COVID-19…”

*(Page 17; line 389)*

“Our result determined that all non-pharmaceutical interventions, including the closure of schools^b^..”

*(Page 19; line 454-457)*

“However, when the number of cases increased with the apparition of new clusters, coordination between centralized isolation and non-pharmaceutical interventions would facilitate controlling the crisis efficiently.”

**Comment 10)** The figures are a little bit blurred in the review version, but this may be due to graphics compression during the processing of the manuscript?!

Author Response: Dear Reviewer, thank you so much for this remark. As you said, it might be due to the graphics compression during the processing of the manuscript. However, we have adjusted the resolution of our figures to achieve a better visualization. Please recheck it and I hope it responses to your requirement.
